# Cognitive Dysfunction in Patients Treated with Androgen Deprivation Therapy: A Multimodality Functional Imaging Study to Evaluate Neuroinflammation

**DOI:** 10.1155/2023/6641707

**Published:** 2023-10-18

**Authors:** Azeem Saleem, Syed Imran Ali Shah, Stephen A. Mangar, Christopher Coello, Matthew B. Wall, Gaia Rizzo, Terry Jones, Patricia M. Price

**Affiliations:** ^1^Invicro, Burlington Danes Building, Imperial College London, Hammersmith Hospital, Du Cane Road, London, UK; ^2^Hull York Medical School, University of Hull, Cottingham Road, Hull HU6 7RX, UK; ^3^Department of Surgery and Cancer, Imperial College, London, UK; ^4^Department of Biochemistry, CMH Lahore Medical College & Institute of Dentistry, Lahore, Pakistan; ^5^Imperial Urology, Imperial College Healthcare NHS Trust, London, UK; ^6^Division of Brain Sciences, Imperial College London, London, UK; ^7^Department of Radiology, University of California Davis Medical Center, Davis, California, USA

## Abstract

**Background:**

Androgen deprivation therapy (ADT) for prostate cancer is implicated as a possible cause of cognitive impairment (CI). CI in dementia and Alzheimer's disease is associated with neuroinflammation. In this study, we investigated a potential role of neuroinflammation in ADT-related CI.

**Methods:**

Patients with prostate cancer on ADT for ≥3 months were categorized as having ADT-emergent CI or normal cognition (NC) based on self-report at interview. Neuroinflammation was evaluated using positron emission tomography (PET) with the translocator protein (TSPO) radioligand [^11^C]-PBR28. [^11^C]-PBR28 uptake in various brain regions was quantified as standardized uptake value (SUVR, normalized to cerebellum) and related to blood oxygen level-dependent functional magnetic resonance imaging (BOLD-fMRI) choice-reaction time task (CRT) activation maps.

**Results:**

Eleven patients underwent PET: four with reported CI (rCI), six with reported NC (rNC), and one status unrecorded. PET did not reveal any between-group differences in SUVR regionally or globally. There was no difference between groups on brain activation to the CRT. Regardless of the reported cognitive status, there was strong correlation between PET-TSPO signal and CRT activation in the hippocampus, amygdala, and medial cortex.

**Conclusions:**

We found no difference in neuroinflammation measured by PET-TSPO between patients with rCI and rNC. However, we speculate that the strong correlation between TSPO uptake and BOLD-fMRI activation in brain regions involved in memory and known to have high androgen-receptor expression mediating plasticity (hippocampus and amygdala) might reflect inflammatory effects of ADT with compensatory upregulated/increased synaptic functions. Further studies of this imaging readout are warranted to investigate ADT-related CI.

## 1. Introduction

Cancer-related cognitive impairment is a common problem with various aetiologies including biological, psychosocial, and treatment factors that may differ between cancers and treatments [1]. Investigations into the pathophysiology of cancer-related cognitive impairment are important for cancer patients and their families and carers and for understanding pathophysiological processes and potential mitigation or treatment. In prostate cancer, a possible association between cognitive impairment (CI) and androgen deprivation therapy (ADT) is an increasing focus of research [2–5]. Androgens are known to have neuroprotective properties, and it is hypothesized that the link between ADT and CI is due to reduced androgen activity in the brain [6]. The extent of CI with ADT is controversial with some studies reporting no association and others reporting prevalence rates of up to 69% [2–5, 7–9]. There is also growing evidence to show that ADT is associated with dementia and Alzheimer's disease, although not all studies support this association [8–12]. There is scant literature on the reversibility of cognitive dysfunction on discontinuing ADT. Clinical recognition of CI after initiating ADT may be through patient reported outcomes and/or psychometric testing, which owing to lack of sensitivity may not capture the incidence and extent of cognitive changes in all patients. Prostate cancer itself seems to have little effect on cognitive performance before treatment [13].

Neuroimaging provides several ways to study cognitive impairment [14], offering the potential to provide new evidence on the question of CI in ADT and to explore new approaches for detecting ADT-related CI in the clinic. Neuroimaging studies have confirmed the presence of neuroinflammation in dementia, Alzheimer's disease, and in patients with mild cognitive impairment [15, 16]. Neuroinflammation is characterized by local activation of glial cells in the brain, which release proinflammatory factors including tumor necrosis factor-*α*, interleukin-1*β*, free radicals, and eicosanoids [17]. Positron emission tomography (PET) imaging can be used to detect and quantify the activation of microglia using a tracer ligand specific for the translocator protein 18 kDa (TSPO), which becomes overexpressed upon activation of microglial cells [16, 18–21]. Despite the evidence of the role of neuroinflammation in dementia, Alzheimer's disease, and mild cognitive impairment, there is a paucity of evidence on the potential role of neuroinflammation in CI associated with ADT.

In this study, we investigated a potential role of neuroinflammation in CI associated with ADT. We conducted PET scans using the TSPO radioligand [^11^C]-PBR28 to assess neuroinflammation in patients on ADT with and without reported CI (rCI) based on detailed medical history. We also conducted blood oxygen level dependent-functional magnetic resonance imaging (BOLD-fMRI) to investigate possible anatomical areas which may be activated in ADT-induced CI in response to cognitive tasks.

## 2. Patients and Methods

### 2.1. Study Population and Screening Procedures

Eligible patients were between 50 and 80 years of age on ADT with luteinizing hormone releasing hormone agonists (LHRHa) for 3–12 months. Blood was sampled at screening to determine TSPO genotype at residue 147 (single nucleotide polymorphism rs6971), with homozygosity for alanine indicating high affinity for synthetic TSPO ligands. High affinity binding was an inclusion criterion. Patients with a known history of organic brain disorders and associated dementia, delirium, and other specific neuropsychiatric conditions, including stroke and head injury and those who were clinically assessed as having mild cognitive impairment before starting their ADT were excluded from the study. Patients who satisfied the eligibility criteria at screening then underwent detailed neurocognitive testing, [^11^C]-PBR28 PET-computed tomography and magnetic resonance imaging.

The study initially planned to recruit 12 age-matched patients on LHRHa stratified to two groups: with ADT-emergent CI based on self/family identification through interview (reported cognitive impairment; rCI), and without (reported normal cognition; rNC). Study results were reviewed periodically, and an adaptive design was used to tailor recruitment cohorts during the study. Based on interim data results after 9 patients, 2 patients were further recruited to undergo dynamic scanning with arterial blood sampling and radio-metabolite analysis to exclude any potential interaction between ADT and [^11^C]-PBR28 that may have an impact on the cerebral uptake of [^11^C]-PBR28 and confound the results. The number of patients included in this pilot study was based on feasibility and pragmatic considerations in this group of patients with prostate cancer, rather than on formal statistical power calculations. Between ten and twelve patients were considered sufficient to provide an initial evaluation of the potential of PET-TSPO imaging to explore the imputed role of ADT in CI.

### 2.2. Study Procedures

#### 2.2.1. Neurocognitive Testing

Neurocognitive functions were assessed using the following paper-based standard neuropsychological questionnaires: Wechsler Abbreviated Scale of Intelligence similarities and matrix reasoning subtests for current verbal and nonverbal reasoning ability; verbal fluency, letter fluency, and color-word (Stroop) tests from the Delis–Kaplan Executive Function System to assess cognitive flexibility, inhibition, and set shifting; Trail Making Test (forms A and B) to assess executive functions; digit span subtest of the Wechsler Memory Scale, third edition (WMS-III) for working memory; and logical memory I and II subtests of the WMS-III as measures of immediate and delayed verbal recall.

#### 2.2.2. PET Imaging Procedure

All PET scans were performed in an outpatient setting at the Invicro Imaging Centre (known as Imanova at the time of the study), London. Scans were performed on Siemens PET-CT scanners (two similar scanners used: Hi-Rez Biograph 6 and Biograph 6 TruePoint with TrueV, Siemens Healthcare, Erlangen, Germany). Patients underwent PET imaging of their brain after intravenous administration of [^11^C]-PBR28. Radiosynthesis and preparation of [^11^C]-PBR28 to good manufacturing practice (GMP) for clinical use was carried out as described previously [22]. PET emission data were acquired in list-mode for 90 minutes (frame durations: 8 × 15 s, 3 × 60 s, 5 × 120 s, 5 × 300 s, 5 × 600 s). A low dose CT of the head was performed just before the PET acquisition to estimate tissue attenuation. PET images were reconstructed using Fourier rebinning and a 2D filtered discrete inverse Fourier transform algorithm with 5 mm isotropic Gaussian filter on a 128 × 128 matrix with 2.6 zoom. Corrections were applied for attenuation, random, and scatter coincidences.

In the 2 patients who had dynamic PET scan with radial arterial blood sampling, whole blood activity data were obtained from a continuous sampling system (Allogg AB, Mariefred, Sweden), measured at a frequency of 1 Hz for the first 15 minutes. Manual (discrete) blood samples were withdrawn at the following time points: 20, 25, 30, 40, 50, 60, 70, 80, and 90 minutes after scan start. Three additional blood samples were withdrawn for calibration purposes at 5, 10, and 15 minutes after scan start. Discrete samples were analyzed for radioactivity in whole blood and plasma components using a Perkin Elmer (Cambridge, UK) Wizard 1470 gamma counter. A subset of the discrete plasma samples (5, 10, 20, 30, 50, 70, and 90 minutes) also underwent analysis to determine the fraction of radioactivity corresponding to the intact parent radiotracer compound, as opposed to radioactive metabolites.

#### 2.2.3. Magnetic Resonance Imaging

All 9 patients in the initial cohort had structural and functional MRI scans on the same day of the PET scan. Structural MRI scans were performed using 3T scanners (Siemens Healthcare, Erlangen, Germany). The MRI sequences included structural (T1-weighted) imaging data which were acquired in the sagittal plane, utilizing a 3D magnetization prepared rapid gradient echo scan with the following parameters: repetition time = 2300 ms, echo time = 2.98 ms, flip angle = 9°, isotropic voxels = 1.0 mm × 1.0 mm × 1.0 mm, 160 slices, and total scanning time = 5 min, 3 sec. Scans were reviewed by a neuroradiologist to exclude any clinically relevant brain abnormalities. T1 MRI data were used as part of the PET data analysis as follows: functional data were acquired with a standard gradient-echo, echo-planar imaging sequence for BOLD contrast, with 3 × 3 × 3 mm voxels, 35 axial slices, a field of view of 192 mm, TE = 30 ms, TR = 2000 ms, in-plane acceleration (GRAPPA) = 2, flip angle = 80°, and bandwidth = 1906 Hz/pixel. The duration of the CRT task was five minutes and 20 seconds, and 161 volumes were acquired. The task was a simple block-design with task blocks lasting 31.5 s, consisting of a series of trials. On each trial a left or right-facing arrow was presented on the screen, and patients responded with a left or right button-press on an MRI-compatible response pad. Patients were given 1.2 s to respond on each trial, and trials were separated by an intertrial interval of 1.75 s. Task blocks were separated by baseline/rest blocks of a similar length and there were five cycles of task/rest blocks in total.

### 2.3. Data Analysis

#### 2.3.1. PET Data Analysis

PET image data were analyzed using Invicro's in-house code, now included in the MIAKAT™ software package. MIAKAT™ is implemented using MATLAB (version R2016a; Mathworks Inc., Natick, MA, USA) and makes use of FSL (version 5.0.4; FMRIB, Oxford, UK [23]) functions for brain extraction and SPM12 (Wellcome Trust Centre for Neuroimaging, https://www.fil.ion.ucl.ac.uk/spm) for image segmentation and registration.

Dynamic PET images registered to each patient's MRI scan (where available) were corrected for motion using a frame-to-frame registration process with a normalized mutual information cost function. For patients in whom a MRI scan was acquired as part of the study (*n* = 9), patient's structural MRI image underwent brain extraction and gray matter segmentation and was coregistered to a standard reference space (MNI152) [24]. The MNI152 template brain image and associated atlas (CIC atlas [25]) was nonlinearly warped to the patient's MRI image to enable automated definition of regions of interest. Regions of interest were defined on the frontal lobe, occipital lobe, temporal lobe, parietal lobe, hippocampus, amygdala and cerebellum (as representative cortical regions), and striatum and thalamus as subcortical regions. Additional larger regions were included such as cortex (average of occipital, temporal, frontal, parietal, insular cortex, hippocampus, and amygdala), subcortex (average of pallidum, striatum, and thalamus), as derived by the CIC atlas definition, and “whole brain,” defined as the union of all the regions in the atlas, i.e., covering the whole brain volume. All regions were gray matter masked, except thalamus, hippocampus and amygdala. The cerebellum was used as pseudoreference region as described previously [26].

For patients in whom a MRI scan was not performed (*n* = 2; patients 106 and 107), the atlas derivation process was slightly different, and a PET-based normalization of the atlas was implemented. Briefly, the individual PET was normalized to the PET image of one of the patients with MRI (therefore of a patient where there was an atlas defined in single patient space using its MRI). Then, the CIC atlas was mapped onto the individual PET using the inverse transformation. The atlases derived in this way were satisfactory with reasonable regions definition, but since this approach does not rely on any fine structural detail of the MRI, results need to be taken with caution in comparison with other groups. For this reason, results for the two patients with unknown cognitive status are discussed separately, focusing on big regions, with a focus to test whether there was an effect on peripheral metabolism of the radioligand. Regions were defined as cortex (average of occipital, temporal, frontal, parietal, insular cortex, hippocampus, and amygdala), subcortex (average of pallidum, striatum, and thalamus), and cerebellum to be used as reference region. A whole brain region covering the whole brain volume was also derived.

The regions of interest were applied to the dynamic PET data to derive regional time–activity curves, with activity concentrations expressed as standardized uptake values (SUV), normalized by injected activity and patient's bodyweight. Outcome parameters were SUV values (g/ml) calculated between 60 and 90 minutes. SUV normalized to the pseudoreference region, cerebellum [26], was also calculated for the same time interval (SUVR).

For the two patients who underwent arterial blood sampling, continuous blood data were calibrated to match overlapping discrete whole blood samples measured at 5, 10, and 15 minutes and then merged with the remaining discrete whole blood data to form a whole blood activity curve covering the duration of the scan. Activity measurements from the discrete plasma samples were divided by the corresponding whole blood data to form plasma-over-blood data. Plasma-over-blood data were fitted to an “exponential approach to a constant curve.” The resulting fitted plasma-over-blood profile was multiplied by the whole blood curve to give a total plasma curve. Parent fraction (metabolite) data were calculated from the high-performance liquid chromatography data and then fitted to a “sigmoid-2” curve. The resulting fitted parent fraction profile was multiplied by the total plasma curve (calculated as described above) and then smoothed postpeak using a tri-exponential fit to give the required parent plasma input function. For each scan, a time delay was fitted and applied to the input function to account for the separation between blood sample measurement and tissue of interest. Time–activity curves were analyzed using the reversible 2-tissue compartmental model with fixed blood volume (to 5%). The main outcome of the 2-tissue compartmental model is the total volume of distribution (*V*_*T*_). *V*_*T*_ values for the two patients were compared with the values published in the literature in age-matched studies [22, 26, 27].

#### 2.3.2. Functional MRI Analysis

All processing and analysis of the functional images was performed using FSL version 5.0 (Oxford, UK). Preprocessing of the CRT task functional data involved removal of nonbrain tissue, head-motion correction, spatial smoothing with a 6 mm full-width-half-maximum Gaussian kernel, and high-pass temporal filtering with a cut-off of 100 s. For the first-level statistical analysis, models were constructed with a single regressor of interest derived from the onset and offset times of the task blocks and also included six head-motion (three translation and three rotation) regressors derived from the preprocessing step. Autocorrelation correction was performed with FSL's FILM algorithm. Group-level analysis used FSL's mixed-effects FLAME-1 model for a robust treatment of both within and between-patient variability and computed a single contrast of CRT > Rest. Thresholding was performed using a cluster-based correction method (*Z* = 2.3, *p* < 0.05) to correct for multiple comparisons.

#### 2.3.3. Statistical Testing

In this small exploratory study, it was not expected that statistically significant differences would be evident in clinical measures between the rCI and rNC groups. Nevertheless, between-group statistical testing of cognitive function tests was conducted using Student's *t* test. Whole-cohort correlations between CRT activation and radioligand uptake on fMRI were determined using Pearson's method.

## 3. Results

### 3.1. Patients and Neurocognitive Assessment

Eleven patients were recruited to the study and underwent imaging (Table 1). Mean age was 68.5 years (SD 7.5). All patients had high-affinity binding TSPO genotype. In the first cohort of nine patients recruited between December 2014 and May 2016, rCI was recorded in four patients and rNC in five patients. In the second cohort of two patients, recruited between August 2017 and October 2018, one patient was recorded as rNC and in the other patient cognitive status was not recorded due to logistical error (only used for reference tissue validity model).

Neurocognitive tests showed numerically lower mean cognitive performance in the rCI group than the rNC group for matrix reasoning, digital span test, logical memory test-retention, color word test (reading), color word test (naming), and verbal fluency test (Table 2). Differences between groups did not reach statistical significance in any of the tests.

### 3.2. PET Imaging

**Figure 1:**
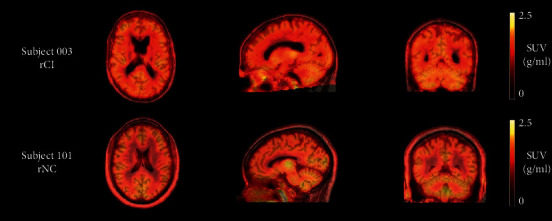
Orthogonal cross sections of coregistered PET and MR images from two representative patients (003 and 101). PET images are shown as SUV summed from 0–90 minutes. Images show regional heterogeneity consistent with the expected distribution of TSPO.

Radioactivity administered ranged 294–374 MBq (mean: 336.8 MBq, SD: 27.6 MBq), with injected masses of [^11^C]-PBR28 ranging from 2.68–8.88 *µ*g (mean: 3.98 *µ*g, SD: 1.95 *µ*g). Visual inspection of PET scans revealed good uptake of the radiotracer in all brain regions. There was no visible trend of increased ligand uptake in rCI patients compared to rNC patients (Figure 1).

**Figure 2:**
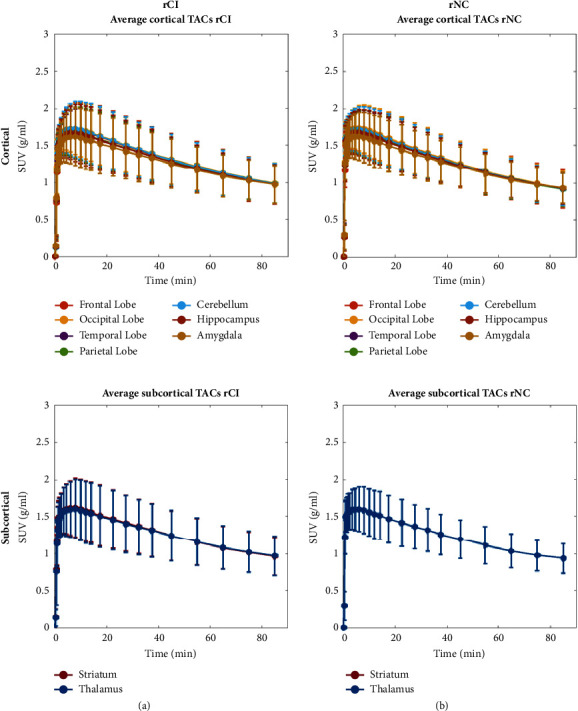
Time–activity curves expressed as SUV for a set of cortical (frontal, occipital, temporal, parietal lobe and cerebellum) and subcortical regions (striatum, thalamus, hippocampus and amygdala), averaged across the rCI and rNC groups. Top and bottom rows report cortical and subcortical regions, respectively. (a) and (b) report rCI and rNC groups, respectively.

PET radiotracer uptake was rapid and very similar in all tissues in the initial minutes, reaching a peak at around 5–10 minutes postinjection, followed by a slow washout. Average time–activity curves across the groups shown in Figure 2 do not show any clear differences between rCI and rNC groups in [^11^C]-PBR28 kinetics in cortical or subcortical regions.

**Figure 3:**
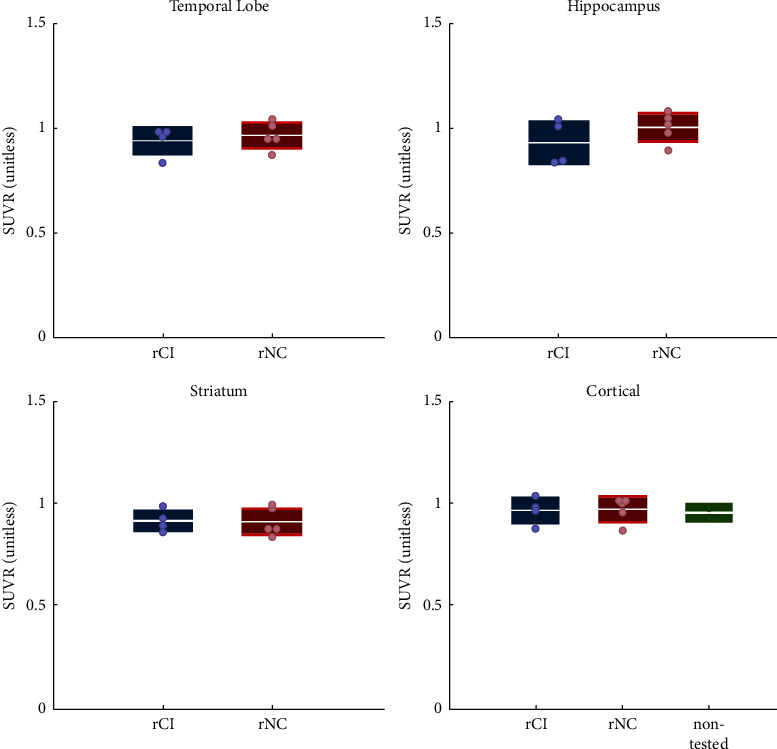
SUVR values in a subset of regions of interest, comparing rCI and rNC in temporal lobe, hippocampus, and striatum. In the bottom right panel rCI, rNC and unknown cognitive status patients SUVR values are compared in a large cortical region, to accommodate for the different spatial preprocessing of the PET images of the patient with unknown cognitive status.

Overall, there were no clear differences in SUV and SUVR values between patients with rCI and rNC (Figure 3, Table S1). PET uptake is reported in the hippocampus, as the region of the neurogenic stem cells, striatum as subcortical region, cerebellum, and finally temporal lobe as these are regions with generally higher [^11^C]-PBR28 PET signal in Alzheimer's disease [28]. Individual SUV and SUVR values for each patient in all the regions analyzed are given in Tables S2 and S3, respectively.

Since the PET images of the patients who underwent dynamic imaging with arterial blood sampling did not have a MRI for spatial processing, the results are presented separately, focusing on some major regions, but overall there were no significant differences in either SUV or SUVR in these two patients compared to rCI or rNC (average SUV for patients in cortex and subcortex: 0.74 ± 0.11 and 0.78 ± 0.04, respectively, average SUVR: in cortex and subcortex: 1.00 ± 0.05 and 0.96 ± 0.03, respectively), in agreement with the results in Table S1.  Table S4 reports the SUV, SUVR, and *V*_*T*_ for the patients who had dynamic imaging with blood sampling and metabolite analysis. Although the SUV values for the two patients without MRI were lower in both cortex and subcortex (Table S4) compared with the other patients, the SUVR values obtained after normalization with a pseudoreference region were similar to the other patients' values and likely to be linked to the different spatial preprocessing (i.e., region definition based on a PET atlas) in these patients. Regional *V*_*T*_ values obtained in this small cohort (Table S4) were in line with previously published results [22, 26], ruling out a systemic effect of LHRHa on brain radioligand uptake.

### 3.3. Functional MR Imaging

Group-level activation maps (all patients) for the CRT task showed a predicted pattern of brain regions responding to the task demands, including the cerebellum, primary visual cortex, motor cortex, and parietal and frontal areas often referred to as the dorsal attention network (e.g., the frontal eye fields) (Figure S1). The areas identified in this analysis were used to produce a single region-of-interest mask (representing the brain network responding to the task) in order to compare these results with the PET data. There were no evident differences between the groups in their BOLD response data within this network-level region of interest.

**Figure 4:**
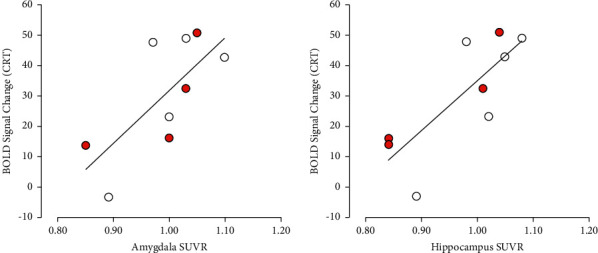
Analysis of SUVR values vs BOLD signal change in a CRT task in the regions showing significant correlation (*p* < 0.05). Red circles indicate rCI patients, white circles indicate rNC patients.

Interestingly, we found strong and significant correlations between CRT activation maps and radioligand uptake (SUVR) in the hippocampus (*R* = 0.72, *p* = 0.03) and amygdala (*R* = 0.79, *p* = 0.01) (Figure 4). The full results from correlations of the CRT task data with SUVR PET values (Pearson's correlation coefficients and relative *p* values) are reported in Table S5.

## 4. Discussion

Our hypothesis for PET imaging was that TSPO, a biomarker of neuroinflammation, will be overexpressed in the brain of patients with CI on ADT. We however did not detect any differences in either global or regional uptake (SUV) of the TSPO radioligand [^11^C]-PBR28 or when normalized to a pseudoreference region (SUVR) between patients with rCI, rNC, or those not tested for cognition. We noted that uptake (SUVR) of [^11^C]-PBR28 in whole brain obtained in our cohort of patients with rCI (0.97–1.03) was similar to brain uptake in similarly aged CI patients by Lyoo et al. where the mean age was 72 years and SUVR was 0.99–1 [26]. Similarly, we did not observe any significant differences in distribution volume patterns in patients on ADT compared to literature data of healthy volunteers [22, 27], with the *V*_*T*_ in our 2 patients (3.99 ± 0.12 ml/cm^3^) being similar to that obtained by Owen et al. who observed a mean *V*_*T*_ of 4.33 ± 0.29 ml/cm^3^ in their study, in a healthy cohort of high affinity binders, aged between 20 and 63 years (42 ± 15 years) [22]. Varnäs et al. also reported similar results for both SUV and *V*_*T*_ in their healthy cohort of high affinity binders, age between 56 and 72 years (brain SUV: 1.1 ± 0.23, brain *V*_*T*_: 3.1 ± 0.54 ml/cm^3^) [27]. The absence of increased [^11^C]-PBR28 uptake either globally or regionally in the brain of patients on ADT or any difference in uptake between patients with or without rCI indicates the absence of TSPO overexpression, and by implication, the absence of neuronal activity or microglial activation in patients on ADT, irrespective of their cognitive status.

Although we found no difference in [^11^C]-PBR28 uptake between patients with rCI and rNC, we did observe strong correlation between CRT activation maps and [^11^C]-PBR28 uptake in the hippocampus and amygdala, regardless of reported cognitive status. The hippocampus and amygdala are both areas of high AR expression mediating brain plasticity. The fMRI activation maps show a relatively standard pattern for tasks of this type, with the visual cortex and dorsal attention regions all showing robust activation, reflecting the visual/attentional nature of the tasks. The correlation between CRT activation and [^11^C]-PBR28 uptake in the hippocampus and amygdala raises the hypothesis that these regions of key cognitive importance may be manifesting an early compensatory mechanism in response to reduced AR activation to maintain functionality and perform the task. A reduction in AR signaling in these regions may impair function so that more metabolic resources are required, leading to an increase in the BOLD response when these areas are active. This phenomenon has also been recently observed in Parkinson's disease [29], and amnestic mild CI [30]. Although speculative, this compensatory activity may also be an attempt to respond to the reduced serum levels of testosterone, as seen preclinically [31–33]. As all subjects in our study were patients with prostate cancer on ADT, it was not possible to distinguish whether this correlation between CRT activation and TSPO uptake is a feature specific to ADT treatment or to prostate cancer. Consequently, this hypothesis warrants further investigation in a larger study that would include age-matched cohorts of prostate cancer patients not on ADT and healthy volunteers. Increased TSPO expression in the hippocampus, amygdala, and thalamus has been implicated with reduced cognitive performance in patients with HIV, and with disease progression in Alzheimer's disease [34, 35].

Besides being rich in AR, the hippocampus is a highly plastic structure that plays a key role in processing higher order information and that also retains the ability to produce new neurons [36, 37]. The key role the hippocampus plays in cognition is well recognized and special consideration is made in several medical disciplines such as radiotherapy, where hippocampal sparing of radiotherapy has resulted in preservation of cognitive function [38]. Our observations support the plasticity of the hippocampus in our patients who at the time of imaging had normal cognition or were reported to have cognitive impairment.

Although studies have been equivocal, there is growing evidence to suggest that ADT affects cognition in some but not all men. This may be a consequence of testosterone suppression, as cognitive changes are associated with lower free testosterone levels especially in those >70 years old [39]; this is also likely to confound the difficulty in assessing the association between ADT and cognitive changes as a significant number of men on ADT are of an older age group. Lower levels of testosterone may also be a plausible explanation for the reported growing evidence that dementia and Alzheimer's disease are associated with ADT. Androgen receptors (ARs) are widely expressed in the brain, with the hippocampus and amygdala, areas also associated with memory, emotional processing, and libido, showing the greatest expression of AR [40, 41]. Androgen deprivation is also known to cause significant loss of hippocampal synapses in rodents and nonhuman primates and increase amyloid deposition in human and rodent models [41]. Postulated mechanisms by which low testosterone levels lead to cognitive changes include increasing serum and brain amyloid-beta levels leading to accumulation of abnormally folded *β*-amyloid [42–45], a feature characteristic of Alzheimer's disease [46], fostering a proinflammatory environment leading to loss of neuroprotection [47–49], and impaired axonal regeneration [50].

In this study, we sought to understand the underlying pathophysiological processes using multimodality functional imaging techniques in patients on ADT. Previous fMRI studies have shown reduced, task-related BOLD-fMRI activation in patients on ADT compared with controls [51], and patients on ADT have demonstrated decreased medial prefrontal cortical activation and decreased connectivity was seen between the medial prefrontal cortex and other regions involved with cognitive control compared to prostate cancer patients not on ADT [52], PET imaging studies with fluorodeoxyglucose (FDG), a glucose analog radiotracer of glycolytic activity in patients on ADT has also revealed decreased regional cerebral glucose metabolism bilaterally in the cerebellum, posterior cingulate, and medial thalamus, regions with metabolic decline found in early Alzheimer's disease and diabetes [53]. To the best of our knowledge, this is the first study that has utilized PET and fMRI, two complementary functional imaging readouts, in the same patients to further understand the pathophysiology of cognitive dysfunction in prostate cancer patients on ADT.

For PET imaging, we chose to use the TSPO radioligand, [^11^C]-PBR28, which has been used as a surrogate PET biomarker of neuroinflammation [19, 22, 26, 27]. However, TSPO is an evolutionarily conserved protein localized primarily in the outer mitochondrial membrane and is involved in a variety of key cellular physiological processes such as steroidogenesis, protein import, cellular proliferation, immunomodulation, regulation of mitochondrial metabolism, and cellular oxidative process [54]. Although, TSPO overexpression has been used as a marker of microglial activity in the brain, TSPO protein is present in neuronal and non-neuronal cells of cortical and subcortical brain regions and increased neuronal activity has demonstrated consistent increases in TSPO gene and protein levels in neurons but not microglia [55]. Therefore, increased TSPO expression may represent not only neuroinflammation but also noninflammatory processes.

TSPO also plays an integral role in the formation of steroid hormones such as testosterone in a variety of organs including the brain by mobilizing and transporting cholesterol into the mitochondria [31]. Studies have shown that circulating and tissue steroid levels are significantly affected in a hormone-independent manner when TSPO drug ligands are administered and it is thought to be due to increase in circulating corticosteroid levels in hypophysectomized animals [32], and the pharmacologic activation of TSPO in aged cells and aged animals leading to increased testosterone formation and circulating levels [33]. However, there are no data currently available that provide information on the expression of TSPO in the brain of patients of prostate cancer with and without ADT.

We evaluated the brain expression of TSPO in patients on ADT and related this to BOLD-fMRI activation maps in the same patients. We classified patients into groups with rCI or rNC based on interview, and patients in the rCI group showed numerically lower performance in some but not all subsequent cognitive tests, although the differences were not statistically significant. Previous studies that have used neurocognitive assessments to assess cognitive status have produced variable results and a meta-analysis has shown that patients on ADT do significantly worse on visuomotor tasks compared with control groups, consistent with the known effects of testosterone on cognitive functioning in healthy men [2].

The absence of differential TSPO expression between rCI and rNC patients as initially hypothesized may be due to several possible reasons including the insufficiency of interview to capture the true incidence of cognitive impairment and/or the imputed difference in TSPO expression being too small to detect in a study this size. Other possible reasons include normalization of TSPO expression after an initial increase as seen after traumatic brain injury, where TSPO gene expression increases at 7 days after but returns to baseline levels at 28 days [56] and reduced binding of the radioligand to TSPO, as LHRHa may negate the multifold increase TSPO binding seen in the presence of gonadotrophins [57]. Alternatively, our hypothesis was not true.

However, the key finding of a relationship between TSPO expression in certain brain regions and BOLD-fMRI has provided valuable information on the pathophysiologic processes in the brain of patients on ADT and allowed us to generate a hypothesis on the pathophysiology of cognitive dysfunction in patients on ADT using functional imaging.

Our conclusions based on imaging readouts are however unable to provide further patho-physiologic detail at a cellular level. Further conclusions of the pathophysiology at the cellular level are also limited by the scientific evidence accrued so far, which is still unclear on what TSPO expression indicates and especially in a person receiving therapy that suppresses the production of a steroid hormone. This study also does not provide any information on the exact mechanism for cognitive changes seen in patients on ADT, which may result from an alternative yet complementary mechanism such as changes in cerebral glucose metabolism [53], loss of synaptic plasticity, and the deposition of neurofibrillary proteins (tau 3 and tau 4) in the hippocampus, as observed preclinically [58].

In conclusion, we did not find differences in TSPO expression between patients on ADT with or without reported cognitive impairment. We did however find that TSPO expression in the hippocampus and amygdala was significantly correlated with BOLD-fMRI activation in these areas, suggestive of plasticity in these regions to maintain function. We propose that this should be evaluated further in a larger study as this is likely to provide information on the potential reversibility of cognitive impairment, the role of agents such as memantine in the prevention of cognitive dysfunction [59] in patients on ADT and help in the rational development of cognitive-sparing ADT or cognitive-protective agents.

## Figures and Tables

**Table 1:** Demographic, cognitive status, and imaging information.

Patient number	Age (years)	Body weight (kg)	TSPO genotype	Reported cognitive status	fMRI (CRT) scanning
001	77	71.9	High affinity	rCI	Performed
002	59	99.7	High affinity	rCI	Performed
003	74	75.3	High affinity	rCI	Performed
004	78	122.7	High affinity	rCI	Performed
101	57	77.0	High affinity	rNC	Performed
102	65	95.9	High affinity	rNC	Performed
103	67	73.9	High affinity	rNC	Performed
104	67	89.9	High affinity	rNC	Performed
105	73	68.7	High affinity	rNC	Performed
106^*∗*^	69	69.4	High affinity	rNC	Not performed
107^*∗*^	68	82.5	High affinity	Not recorded	Not performed

^*∗*^Arterial blood sampling performed throughout scan duration. Weight recorded at the PET visit.

**Table 2:** Results of neurocognitive tests.

Test	rNC (*n* = 6) Mean (SD)	rCI (*n* = 4) Mean (SD)
Similarities Test^*∗*^	36.5 (5.36)	31.3 (5.37)
Matrix reasoning	23.5 (2.81)	18.0 (7.62)
Digital span test	20.7 (4.97)	16.8 (3.77)
Peoples test (recall)	19.5 (6.19)	20 (9.2)
Peoples test (delayed recall)	7 (4.0)	7 (5.8)
Logical memory test–retention	86.6 (22.25)	75.5 (17.46)
Logical memory test (recognition)	23.3 (4.27)	23.5 (4.65)
Color word test (reading)^*∗*^	22.8 (6.65)	25 (1.41)
Color word test (naming)^*∗*^	29.2 (3.31)	38.8 (7.27)
Verbal fluency test	52.8 (13.85)	40.5 (8.74)

Lower score indicates lower performance except for tests marked with an asterisk, in which higher score indicates lower performance.

## Data Availability

The datasets generated and/or analyzed during the current study are available from the corresponding author on reasonable request.

## Abbreviations

[^11^C]-PBR28: Radioligand for translocator protein 18 kDa
ADT: Androgen deprivation therapy
BOLD-fMRI: Blood oxygen level dependent-functional magnetic resonance imaging
CI: Cognitive impairment
CRT: Choice reaction time task
fMRI: Functional magnetic resonance imaging
LHRHa: Luteinizing hormone releasing hormone agonists
MRI: Magnetic resonance imaging
PET: Positron emission tomography
rCI: Reported cognitive impairment
rNC: Reported normal cognition
SUV: Sstandardized uptake value
SUVR: Sstandardized uptake value normalized to cerebellum
TSPO: Translocator protein 18 kDa.

## Supplementary Materials

Supplementary Materials Table S1: Mean (SD) SUV, SUVR for patients with rCI and rNC. Table S2: SUV (g/ml) values calculated in the interval 60 to 90 min. Table S3: SUVR values for each patient calculated between 60 to 90 min. Reference region is the cerebellum (gray matter-masked). Table S4: SUV, SUVR, and V_T_ values for the 2 patients with blood measurement and without MRI. V_T_ calculated with reversible two tissue compartmental model, with blood contribution fixed to 5%. Table S5: Pearson's correlation coefficient and corresponding *p*-value of the SUVR values in relevant brain regions versus the activation maps derived with the CRT, memory and Tower of London tasks. Single asterisks mark significant correlation (*p* < 0.05). Figure S1: activation maps from fMRI analysis for the choice reaction time (CRT) task. Mixed-effects (FLAME-1) analysis, and statistical thresholds were set at *Z* > 2.3, *p* < 0.05 (cluster-corrected for multiple comparisons). The results show motor-related activity in the cerebellum and primary motor cortex, and a set of attention-related regions such as the primary visual cortex, the frontal eye-fields, and parietal regions.
