# Supplementary material for: Cognitive Dysfunction in Patients Treated with Androgen Deprivation Therapy: A Multimodality Functional Imaging Study to Evaluate Neuroinflammation
Source: Prostate Cancer. 2023 Oct 18;2023:6641707. doi: 10.1155/2023/6641707 (PMC10599921; doi:10.1155/2023/6641707)

Supplementary Material

Table S1. Mean (SD) SUV, SUVR for patients with rCI and rNC.

	Cerebellum	Temporal lobe		Striatum		Hippocampus		Subcortex		Cortex	
	SUV	SUV	SUVR	SUV	SUVR	SUV	SUVR	SUV	SUVR	SUV	SUVR
**rCI**	1.10 (0.39)	1.01 (0.27)	0.94 (0.07)	0.99 (0.31)	0.91 (0.05)	0.95 (0.25)	0.93 (0.11)	1.01 (0.32)	0.98 (0.10)	1.05 (0.29)	0.97 (0.07)
**rNC**	1.02 (0.26)	0.98 (0.22)	0.97 (0.07)	0.91 (0.29)	0.90 (0.07)	0.98 (0.18)	1.00 (0.07)	0.97 (0.20)	0.99 (0.07)	0.99 (0.26)	0.97 (0.06)

Table S2. SUV (g/ml) values calculated in the interval 60 to 90 min.

Patient	Whole Brain	Frontal Lobe	Occipital Lobe	Temporal Lobe	Parietal Lobe	Striatum	Thalamus	Hippocampus	Amygdala	Cerebellum	Subcortex	Cortex
**001**	0.83	0.83	0.89	0.85	0.85	0.77	0.78	0.72	0.86	0.87	0.75	0.85
**002**	0.83	0.87	0.84	0.78	0.84	0.80	0.94	0.78	0.80	0.81	0.86	0.84
**003**	1.01	1.04	0.99	1.03	0.97	0.96	1.08	1.05	1.07	1.05	0.97	1.02
**004**	1.48	1.47	1.53	1.39	1.47	1.43	1.61	1.25	1.26	1.67	1.48	1.47
**Mean**	**1.04**	**1.05**	**1.06**	**1.01**	**1.03**	**0.99**	**1.10**	**0.95**	**1.00**	**1.10**	**1.01**	**1.05**
**SD**	**0.31**	**0.29**	**0.32**	**0.27**	**0.30**	**0.31**	**0.36**	**0.25**	**0.21**	**0.39**	**0.32**	**0.29**
**101**	0.99	0.95	0.99	0.96	0.90	0.92	1.21	1.09	1.07	1.10	1.06	0.96
**102**	1.02	1.02	1.03	1.01	0.99	0.92	1.07	1.01	1.01	1.06	0.94	1.02
**103**	0.77	0.78	0.74	0.78	0.73	0.75	0.89	0.81	0.77	0.77	0.80	0.77
**104**	1.38	1.44	1.43	1.33	1.43	1.21	1.37	1.21	1.21	1.40	1.26	1.42
**105**	0.77	0.77	0.80	0.80	0.71	0.75	0.84	0.78	0.82	0.76	0.77	0.78
**Mean**	**0.99**	**0.99**	**1.00**	**0.98**	**0.95**	**0.91**	**1.07**	**0.98**	**0.97**	**1.02**	**0.97**	**0.99**
**SD**	**0.25**	**0.27**	**0.27**	**0.22**	**0.29**	**0.19**	**0.22**	**0.18**	**0.18**	**0.26**	**0.20**	**0.26**

Table S3. SUVR values for each patient calculated between 60 to 90 min. Reference region is the cerebellum (grey matter-masked).

Patient	Whole Brain	Frontal Lobe	Occipital Lobe	Temporal Lobe	Parietal Lobe	Striatum	Thalamus	Hippocampus	Amygdala	Cerebellum	Subcortex	Cortex
**001**	0.96	0.95	1.02	0.98	0.98	0.88	0.90	0.84	1.00	1	0.88	0.98
**002**	1.03	1.07	1.04	0.96	1.03	0.98	1.21	1.01	1.03	1	1.11	1.04
**003**	0.97	0.99	0.94	0.98	0.92	0.91	1.06	1.04	1.05	1	0.95	0.97
**004**	0.89	0.88	0.92	0.83	0.88	0.86	1.08	0.84	0.85	1	0.99	0.88
**Mean**	**0.96**	**0.97**	**0.98**	**0.94**	**0.95**	**0.91**	**1.06**	**0.93**	**0.98**	**1.00**	**0.98**	**0.97**
**SD**	**0.06**	**0.08**	**0.06**	**0.07**	**0.06**	**0.05**	**0.12**	**0.11**	**0.09**	**0.00**	**0.10**	**0.07**
**101**	0.90	0.86	0.90	0.87	0.81	0.83	1.14	1.02	1.00	1	1.00	0.87
**102**	0.96	0.96	0.97	0.95	0.93	0.87	1.03	0.98	0.97	1	0.91	0.96
**103**	1.00	1.02	0.95	1.01	0.94	0.97	1.18	1.08	1.03	1	1.06	1.00
**104**	0.99	1.03	1.02	0.95	1.02	0.87	1.01	0.89	0.89	1	0.93	1.01
**105**	1.01	1.00	1.05	1.04	0.93	0.98	1.12	1.05	1.10	1	1.03	1.02
**Mean**	**0.97**	**0.97**	**0.98**	**0.97**	**0.93**	**0.90**	**1.09**	**1.00**	**1.00**	**1.00**	**0.99**	**0.97**
**SD**	**0.05**	**0.07**	**0.06**	**0.07**	**0.07**	**0.07**	**0.07**	**0.07**	**0.08**	**0.00**	**0.07**	**0.06**

Table S4. SUV, SUVR and V_T values for the 2 patients with blood measurement and without MRI. V_T calculated with reversible two tissue compartmental model, with blood contribution fixed to 5%.

	**SUV**				**SUVR**				**V_T**			
**Patient**	**Brain**	**Cerebellum**	**Subcortex**	**Cortex**	**Brain**	**Cerebellum**	**Subcortex**	**Cortex**	**Brain**	**Cerebellum**	**Subcortex**	**Cortex**
106	0.66	0.72	0.75	0.67	0.92	1	1.04	0.93	4.07	4.28	4.89	4.09
107	0.79	0.84	0.81	0.82	0.94	1	0.96	0.98	3.91	4.10	4.00	4.04
**Mean**	**0.72**	**0.78**	**0.78**	**0.74**	**0.93**	**1.00**	**1.00**	**0.96**	**3.99**	**4.19**	**4.45**	**4.07**
**SD**	**0.09**	**0.09**	**0.04**	**0.11**	**0.02**	**0.00**	**0.05**	**0.03**	**0.12**	**0.13**	**0.63**	**0.03**

Table S5. Pearson's correlation coefficient and corresponding p-value of the SUVR values in relevant brain regions versus the activation maps derived with the CRT, memory and Tower of London tasks. Single asterisks mark significant correlation (p <0.05).

	CRT whole map
Pearson's r	0.321
p-value	0.400
Pearson's r	0.220
p-value	0.569
Pearson's r	0.503
p-value	0.167
Pearson's r	-0.178
p-value	0.646
Pearson's r	-0.113
p-value	0.772
Pearson's r	NaN
p-value	NaN
Pearson's r	0.297
p-value	0.438
Pearson's r	0.531
p-value	0.141
Pearson's r	0.405
p-value	0.279
Pearson's r	0.718*
p-value	0.029
Pearson's r	0.792*
p-value	0.011

Figure S1. Activation maps from fMRI analysis for the choice reaction time (CRT) task. Mixed-effects (FLAME-1) analysis, and statistical thresholds were set at Z >2.3, p <0.05 (cluster-corrected for multiple comparisons). The results show motor-related activity in the cerebellum and primary motor cortex, and a set of attention-related regions such as the primary visual cortex, the frontal eye-fields, and parietal regions.

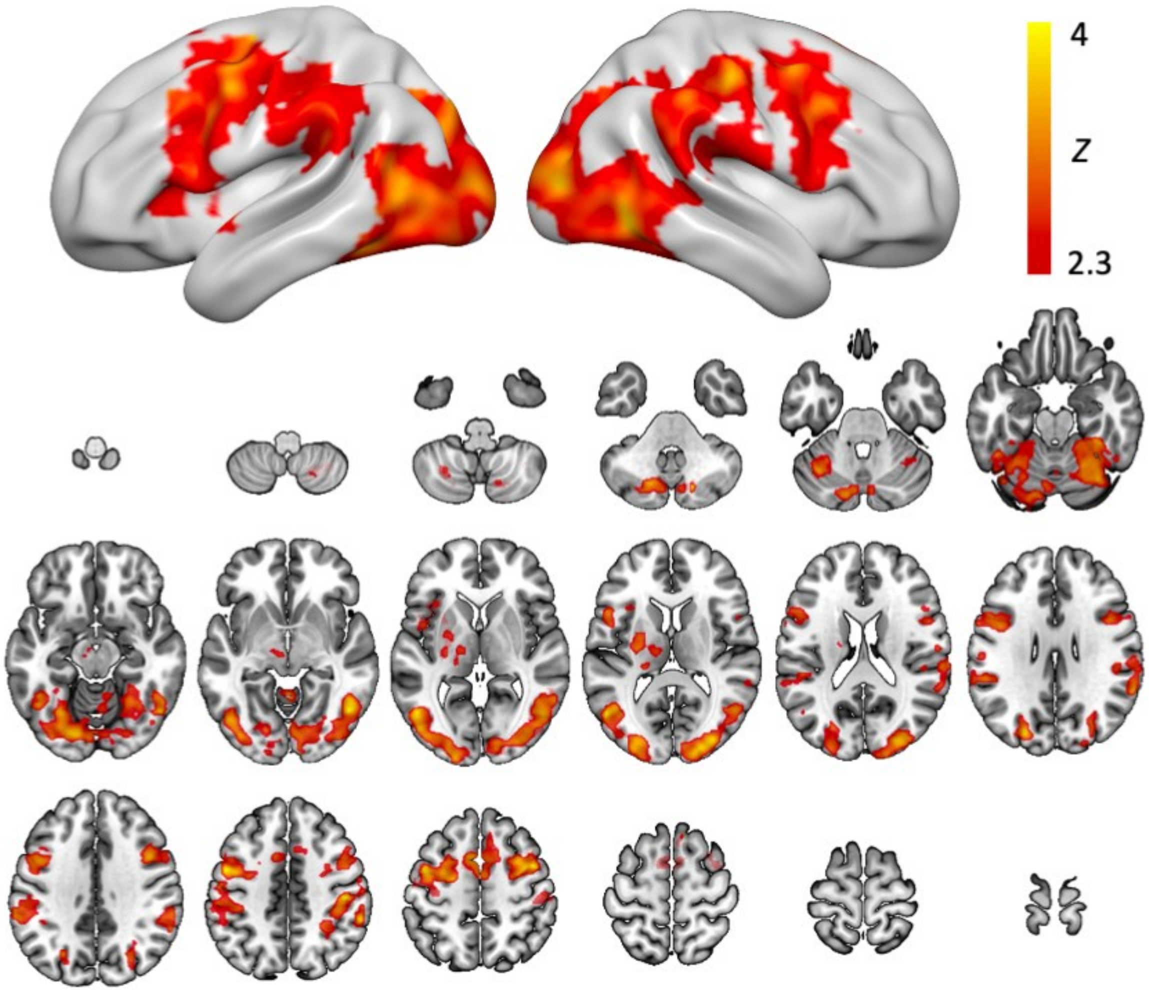

Supplement: Supplementary Materials — Table S1: Mean (SD) SUV, SUVR for patients with rCI and rNC. Table S2: SUV (g/ml) values calculated in the interval 60 to 90 min. Table S3: SUVR values for each patient calculated between 60 to 90 min. Reference region is the cerebellum (gray matter-masked). Table S4: SUV, SUVR, and VT values for the 2 patients with blood measurement and without MRI. VT calculated with reversible two tissue compartmental model, with blood contribution fixed to 5%. Table S5: Pearson's correlation coefficient and corresponding p-value of the SUVR values in relevant brain regions versus the activation maps derived with the CRT, memory and Tower of London tasks. Single asterisks mark significant correlation (p < 0.05). Figure S1: activation maps from fMRI analysis for the choice reaction time (CRT) task. Mixed-effects (FLAME-1) analysis, and statistical thresholds were set at Z > 2.3, p < 0.05 (cluster-corrected for multiple comparisons). The results show motor-related activity in the cerebellum and primary motor cortex, and a set of attention-related regions such as the primary visual cortex, the frontal eye-fields, and parietal regions. [file 6641707.f1.pdf]
